# Haematological and molecular responses in patients with chronic ATLL treated with zidovudine and interferon-α

**DOI:** 10.1186/1742-4690-11-S1-O5

**Published:** 2014-01-07

**Authors:** Andrew Hodson, Maria-Antonietta Demontis, Lucy Cook, Nicolas Gillet, Anat Melamed, Charles RM Bangham, Graham P Taylor

**Affiliations:** 1 Section of Infectious Diseases, Wright-Fleming Institute, Imperial College London, London, UK; 2 Department of Immunology, Wright-Fleming Institute, Imperial College London, London, UK

## 

The utility of HTLV-1 proviral load and integration site analysis by both a linker mediated PCR assay based on the Universal VectoretteTM System (Sigma Genosys) and by high through-put sequencing (HTPS) to assess the treatment response of four patients with chronic adult T-cell leukaemia/lymphoma (ATLL) undergoing first line treatment with zidovudine and interferon alpha (ZDV/IFN-α) at the molecular level is described. Diagnosis of chronic ATLL was according to the Shimoyama criteria. All patients were Afro-Caribbean, median age 52 years (range 32-63), three were female. Median lymphocyte count was 8.2x109/L (range 9-23.9) and HTLV-1 proviral load (PVL) was 73.8% (72.6-276.2). All patients gave informed consent and treatment was according to clinic protocol. All patients remain alive with median overall survival of 64 (27 -106) months. A dominant clone was detected by vectorette and HTPS in all patients. Complete haematological response (CHR), defined as normal white cell count and lymphocyte count for one month, was observed in all patients within nine months of starting treatment. Two patients were intolerant of therapy long term and a molecular response was not observed with persistence of the dominant clones on vectorette and by HTPS. In the two patients tolerant of therapy a reduction in HTLV-1 PVL was observed 10-40 months after the CHR but the dominant clone remained detectable by vectorette for a further 20 months on treatment. Two patients have relapsed off treatment and two remain in CHR. Following CHR molecular methods reveal residual disease and predict long-term response.

